# A Rare Case of Mixed Malignant Ovarian Germ Cell Tumor Composed of Immature Teratoma and Yolk Sac Tumor

**DOI:** 10.7759/cureus.82242

**Published:** 2025-04-14

**Authors:** Emiliya Apinova, Andrew S Martin, Nadim Bou Zgheib

**Affiliations:** 1 Obstetrics and Gynecology, West Virginia School of Osteopathic Medicine, Lewisburg, USA; 2 Obstetrics and Gynecology, Holzer Health System, Gallipolis, USA; 3 Gynecologic Oncology, Cabell Huntington Hospital, Marshall University Joan C. Edwards School of Medicine, Huntington, USA

**Keywords:** immature teratoma, malignant ovarian germ cell tumor, mixed ovarian germ cell tumor, ovarian germ cell tumor, yolk sac tumor

## Abstract

Malignant ovarian germ cell tumors (MOGCTs) are rare but aggressive neoplasms, accounting for a small percentage of ovarian cancers, and often affect young women. We present the case of a 23-year-old woman who presented to the clinic believing she was 14.2 weeks pregnant. However, she was found to have elevated beta-human chorionic gonadotropin (β-hCG) levels and a complex left adnexal mass. Initial ultrasound suggested an ovarian tumor, and surgical exploration revealed a large mixed germ cell tumor measuring 36.5 x 27 x 23.9 cm, weighing 9463.2 grams, with metastases to the small bowel, omentum, and peritoneum. Final pathology confirmed a high-grade immature teratoma and yolk sac tumor with a composition of 60% teratoma and 40% yolk sac tumor (YST). The patient underwent comprehensive staging surgery, including omentectomy, bowel resection, and lymphadenectomy, followed by four cycles of bleomycin, etoposide, and cisplatin (BEP) chemotherapy. Unfortunately, the patient has not responded to the standard chemotherapy regimen of BEP and will pursue further treatment through clinical trials. This case highlights the diagnostic challenge of MOGCTs, particularly mixed forms, which may present with nonspecific symptoms. Timely diagnosis and aggressive treatment, including fertility-preserving surgery when possible, are crucial for improving outcomes. Although survival rates are generally favorable, close follow-up is needed due to the potential for relapse, particularly in cases with advanced disease, as seen in our case. This case emphasizes the importance of considering MOGCTs in young women with elevated β-hCG and adnexal masses and the management of this metastatic disease.

## Introduction

The World Health Organization classifies malignant ovarian germ cell tumors (MOGCTs) into dysgerminoma, endodermal sinus tumor, also known as yolk sac tumor (YST), immature teratoma (IT), non-gestational choriocarcinoma, embryonal carcinoma, and mixed germ cell tumors [1]. Mixed malignant ovarian germ cell tumors are characterized by the presence of two or more malignant germ cell components. While these tumors are uncommon, representing about 8% of all germ cell tumors, they are known for their aggressive behavior [2]. These tumors primarily affect adolescents and young women. Patients typically present with lower abdominal or pelvic pain, distension, or a palpable mass. Acute complications, such as ovarian torsion or hemorrhagic rupture, may occur due to rapid tumor growth and can require emergency surgery [3]. The differential includes benign ovarian cysts, ectopic pregnancy, and other neoplasms. Diagnostic evaluation involves ultrasound, MRI, or CT for staging and tumor markers, such as alpha-fetoprotein (AFP), beta-human chorionic gonadotropin (β-hCG), and lactate dehydrogenase (LDH), to help differentiate histologic components [4]. Management consists of fertility-sparing surgery followed by bleomycin, etoposide, and cisplatin (BEP) chemotherapy in most cases. Although early-stage MOGCTs have a favorable prognosis, with 5-year survival exceeding 90%, patients remain at risk for chemotherapy-related toxicity and late relapse [5]. We present the case of a young woman with a mixed malignant germ cell tumor, composed of a yolk sac tumor and immature teratoma, who initially presented with advanced disease while appearing at the clinic for presumed pregnancy evaluation.

## Case presentation

**Figure 1:**
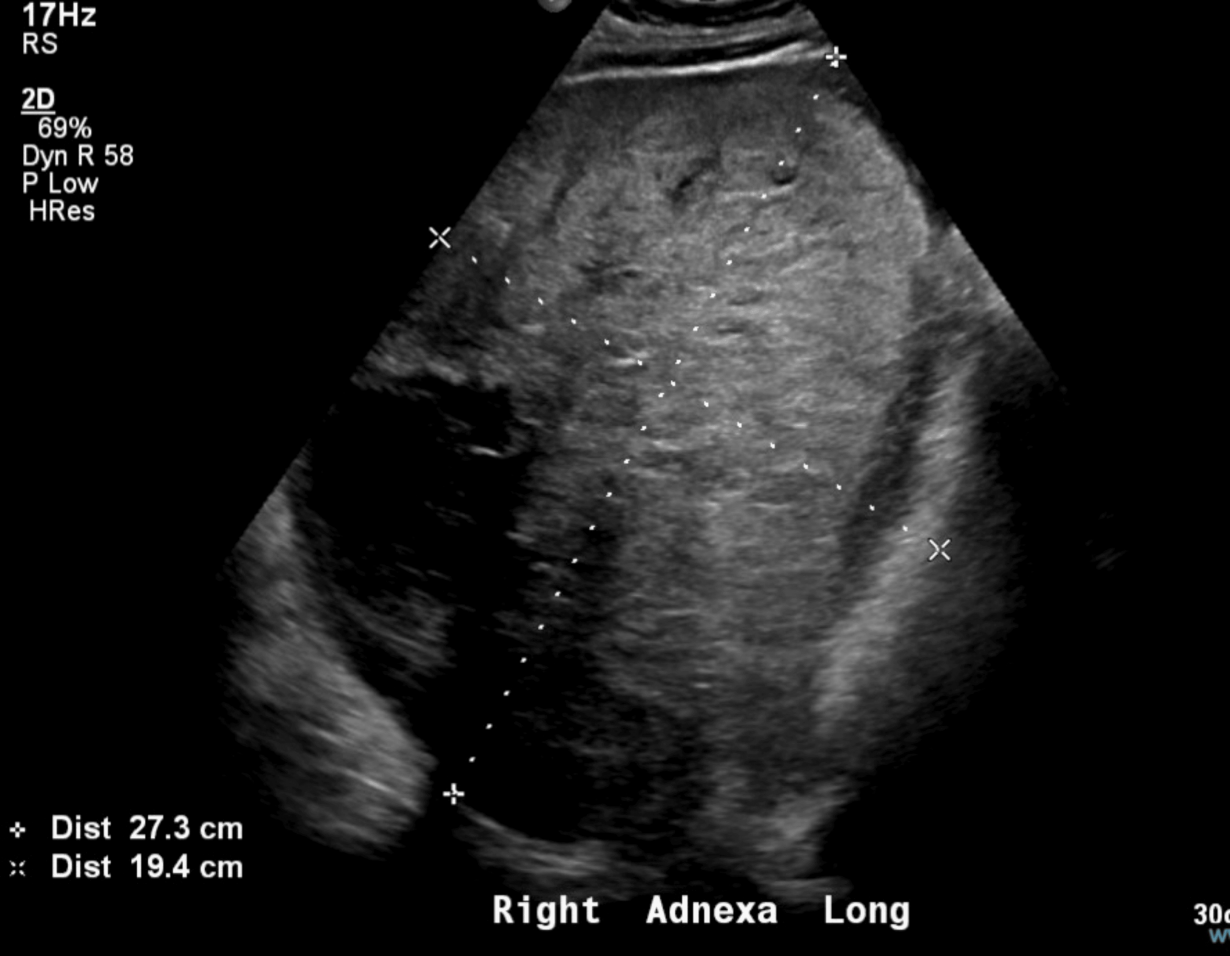
Transvaginal ultrasound of the right adnexa

A 23-year-old nulliparous female presented for pregnancy evaluation following a positive home pregnancy test. The first day of her last menstrual period (LMP) was approximately 2.5 months prior to presentation. She reported mild nausea, but no vomiting, vaginal bleeding, or pain. Her medical history included ovarian cysts, asthma, and obesity. Her family history was notable for a maternal grandmother with breast cancer. Vital signs revealed a pulse of 153/min, oxygen saturation of 97%, and blood pressure of 132/76 mmHg. Ultrasound revealed a normal-sized uterus with a 4-mm endometrial stripe and significant right adnexal masses, including a complex structure measuring 3.1 x 9.7 x 2.3 cm and a solid mass measuring 27.3 x 19.4 x 25.2 cm (Figure 1). Urine tests confirmed elevated beta-human chorionic gonadotropin (β-hCG) levels, which were attributed to a hormone-producing tumor rather than a viable pregnancy.

**Figure 2:**
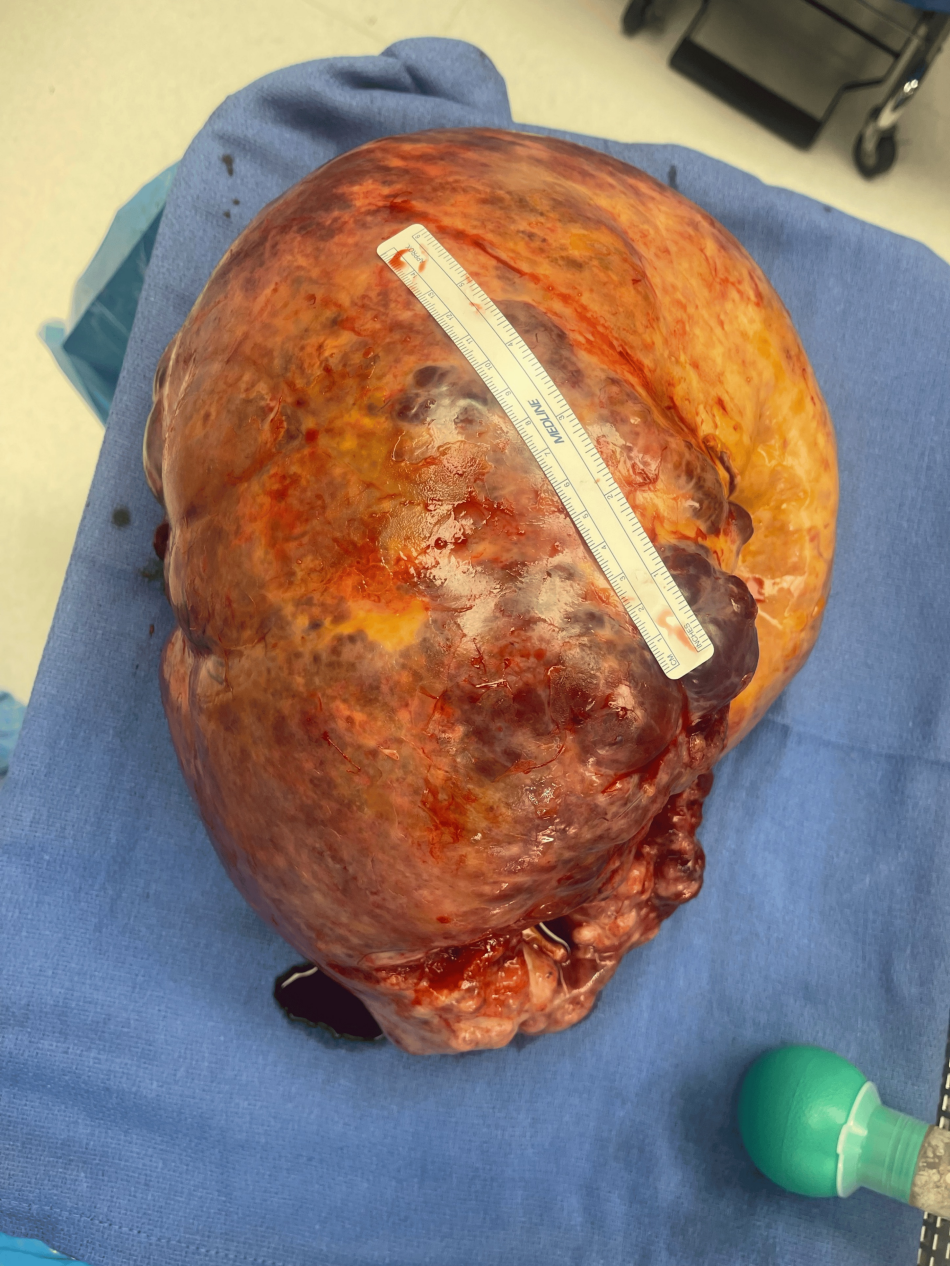
Gross appearance of mixed malignant ovarian germ cell tumor

The patient was referred to a gynecologic oncologist due to suspicion of a germ cell tumor based on elevated β-hCG levels of 79.7 mIU/mL (normal: <5 mIU/mL) and elevated LDH levels of 864 U/L (normal: 140-280 U/L). The patient underwent exploratory laparotomy. Upon entering the peritoneum, ascitic fluid was aspirated for cytology, revealing extensive adhesions that required mobilization of the omentum and surrounding structures. The mass was identified as arising from the left ovary, measuring 36.5 x 27 x 23.9 cm and weighing 9463.2 grams. The right ovary appeared normal. A photograph of the gross tumor is shown in Figure 2.

The utero-ovarian ligaments and the infundibulopelvic ligament were ligated and dissected, leading to the complete excision of the mass, which was sent for pathology. Omentectomy was performed with careful dissection of the omentum from the transverse colon. Extensive tumor nodularity throughout the pelvic and abdominal peritoneum was debulked, including the involvement of small bowel nodules. A segment of the small bowel was resected, with a side-to-side anastomosis completed. Additionally, lymphadenectomy of the right and left pelvic lymph nodes was performed, with all specimens sent for pathological analysis.

The initial pathological staging of the tumor was determined to be pT3bN0M0, indicating that it has extended into the perirenal tissues or renal vein (T3b), with no regional lymph node involvement (N0) and no distant metastasis (M0). Final pathology results revealed a MOGCT involving the left fallopian tube and ovary, small bowel, omentum, cecal region, left peritoneal area, left sigmoid, and both the posterior and anterior pelvic peritoneum. The tumor was characterized as a high-grade (grade 2-3) IT with an intermixed YST component, affecting the ovary, small bowel, sigmoid, cecal serosa, small bowel mesentery, omentum, and peritoneum. Notably, the small bowel mucosa and muscularis propria, as well as the left fallopian tube, were negative for a tumor. Overall, the findings revealed a MOGCT composed of approximately 60% teratoma, including a high-grade immature neuroectodermal component, and 40% YST. Ascitic fluid cytology also tested positive for glypican-3, further confirming the presence of the YST component. The immunohistochemical staining results are summarized in Table 1.

**Table 1:** Immunohistochemical staining results demonstrating a mixed germ cell tumor with positivity for markers consistent with teratomatous, yolk sac, and immature neuroectodermal components. Negative staining for CD30, ER, and GATA-3 helps exclude other tumor types.

Marker	Result
CD30	Essentially negative
CD117	Patchy positive in teratomatous component
Cytokeratin	Positive in teratomatous component
ER	Negative
GATA-3	Negative
GFAP	Positive in immature neuroectodermal component
S100	Positive in foci of teratomatous component
Glypican-3	Positive in yolk sac component
PAX8	Patchy positive

Based on these findings, the patient received adjuvant chemotherapy consisting of four cycles of BEP. Unfortunately, the patient did not respond to the standard BEP chemotherapy regimen. Post-treatment computed tomography imaging revealed extensive mass lesions throughout the liver, a significant 10-cm mass in the porta hepatis, and multiple confluent bulky lesions in the pelvis. These pelvic lesions encircle the uterus and exert displacement and compression on the rectosigmoid region. As a result, the patient is pursuing further treatment through a clinical trial protocol and will be receiving inpatient combination chemotherapy with paclitaxel, ifosfamide, and cisplatin (the TIP regimen).

## Discussion

Pathophysiology

Ovarian malignant germ cell tumors (OGCTs) account for 2.6% of all ovarian cancers, with the highest incidence occurring in the first two decades of life [1]. OGCTs occur due to the pathologic transformation of the primordial germ cell (PGC) during the distinct stages of embryonic development [6]. In MOGCTs, dysgerminoma and YST are the most frequently observed combination [2]. In our case, we identified a rare pairing of IT and YST, which carries a high malignant potential.

IT is the second most common MOGCT, representing less than 1% of ovarian teratomas [7]. It contains tissues from all three germ layers, with immature neuroectodermal tissue being essential for its classification. Grading is based on the amount of this immature neuroectoderm [7]. ITs are typically not linked to increased levels of alpha-fetoprotein (AFP) or β-hCG unless they occur as part of a mixed germ cell tumor, as observed in our case [8]. Diagnosis relies on morphological evaluation as there are no specific immunohistochemical (IHC) markers.

YST is commonly found in pre-menopausal women. YSTs are grossly characterized by large, well-encapsulated, mostly unilateral tumors that have both solid and cystic components, often showing signs of hemorrhage or necrosis [9]. Most patients with YSTs demonstrate elevated serum levels of AFP and cancer antigen-125 (CA-125). The prognosis for YST is significantly influenced by disease stage and elevated tumor markers, particularly AFP, which serves as both a diagnostic and monitoring marker [1]. Distinguishing YST from other tumor types can be challenging; however, IHC markers, such as sal-like protein 4 (SALL4) and glypican-3 (GLP3), are useful for accurate identification [10]. In our case, IHC analysis revealed distinctive staining for AFP, supporting the presence of YST in a mixed germ cell tumor. It is important to note that YSTs are known for their aggressive nature, with a propensity for rapid invasion and metastasis to retroperitoneal lymph nodes and other intra-abdominal structures [2]. Timely diagnosis is critical for optimizing patient prognosis. 

Clinical presentation and diagnosis

The initial evaluation of MOGCTs begins with a thorough medical history and clinical examination, supplemented by imaging techniques such as transvaginal and transabdominal ultrasounds. These tumors primarily affect young women. About 85% present with abdominal pain and a pelvic mass, while 10% may have acute pain resembling appendicitis. Other symptoms include abdominal distention (35%), fever (10%), and vaginal bleeding (10%), with some cases showing premature sexual development linked to β-hCG [1,11]. In some cases, abdominal swelling may occur alongside amenorrhea, which can lead to a mistaken assumption of pregnancy, as demonstrated in our case. Typically, ovarian enlargement is observed on one side; however, there have been cases of bilateral ovarian involvement as well, with its occurrence ranging from 3% to 19% [12].

The different subtypes of MOGCTs are associated with the production of specific serum tumor markers that can be measured in patients' blood, providing valuable diagnostic information. Dysgerminomas are typically associated with elevated levels of LDH and, in some cases, β-hCG. YSTs predominantly produce AFP, which is crucial for diagnosis and management. Additionally, ITs may also elevate AFP levels in about one-third of cases [6]. As seen in our case, elevated β-hCG levels can lead to false-positive pregnancy tests, causing confusion for the patient and complicating the clinical picture. These tumor markers should be evaluated promptly when a MOGCT is suspected, as they are essential to prevent delays in surgical treatment.

Along with serum tumor markers, preoperative indications include the detection of an adnexal mass via examination or pelvic imaging [13]. In our case, the tumor was initially seen on the right ovary via ultrasound; however, during surgery, the mass was found to arise from the left ovary. Extensive adhesions and metastasis can cause ultrasound images to misrepresent tumor location. This can lead to confusion in diagnosis and treatment, emphasizing the need for thorough surgical exploration to accurately identify the tumor's true origin. 

A definitive diagnosis of a malignant mixed ovarian germ cell tumor necessitates histological evaluation, making surgical intervention essential. While conventional histology can often establish a diagnosis, IHC is crucial for confirmation in MOGCTs. Key IHC markers of common ovarian germ cell tumors are listed in Table 2.

**Table 2:** IHC markers for common ovarian germ cell tumors Source: [1] IHC: immunohistochemical

Marker	Dysgerminoma	Yolk Sac Tumor	Embryonal Carcinoma
SALL4	+	+	+
OCT 3/4	+	-	+
CD30	-	-	+
CD117	+	-	-
D2-40	+	-	-
AFP	-	+	+/-
Glypican-3	-	+	-

Surgical management and chemotherapy

Laparotomy is now recommended for the management of MOGCTs due to the tumors' substantial size and solid characteristics [14]. While most germ cell tumors are typically confined to one ovary at diagnosis, careful surgical staging is essential, as occult metastatic disease is often present in the majority of patients. Therefore, patients with ovarian cancer, where the tumor appears macroscopically confined to the ovaries or pelvic area, should undergo thorough surgical staging to evaluate the extent of the disease and guide treatment decisions [15]. The standard recommendation for comprehensive surgical staging includes peritoneal cytology, peritoneal biopsies, omentectomy, and retroperitoneal lymphadenectomy that encompasses bilateral pelvic and para-aortic nodes, along with the excision of any suspicious tissue. In cases of disseminated disease, tumor reductive surgery should be performed [16]. The International Federation of Gynecology and Obstetrics (FIGO) staging system provides surgical guidelines for the classification of carcinoma of the ovary, which aids in determining the extent of the disease and guiding treatment [15,17].

Given that MOGCT predominantly occurs in females of reproductive age, fertility preservation becomes a primary concern. In instances of advanced disease, such as metastasis to the para-aortic lymph nodes, the preservation of reproductive function can still be achieved, especially if the opposite ovary is functioning normally. Routine biopsy of the opposite ovary should be avoided, as it can lead to peritoneal adhesions or ovarian failure, risking future infertility despite few reports of hidden bilaterality [15]. The predominance of unilateral tumors enables fertility-sparing surgery for the majority of patients, with approaches such as unilateral salpingo-oophorectomy being considered to achieve optimal cytoreduction [6]. Cytoreductive surgery offers several benefits such as the removal of tumor masses and ascites, improved delivery of chemotherapy to the tumor, and enhanced immune function in the patient [18].

Surgical management alone is insufficient for treating advanced mixed ovarian germ cell tumors, making it crucial to initiate chemotherapy post-surgery. The treatment of MOGCTs has been successful in gynecologic oncology due to their high sensitivity to chemotherapy. Combination chemotherapy with BEP is a current first-line treatment. Typically, three to four courses of chemotherapy are recommended. In cases of mixed germ cell tumors, additional courses may be considered after tumor markers yield negative results [16]. A key feature of MOGCT is its association with specific serum tumor markers, which are useful for monitoring treatment response during chemotherapy and for ongoing surveillance during follow-up care [19]. Furthermore, studies indicate that patients with metastatic disease who exhibit normal ovarian function prior to treatment do not experience detrimental long-term effects on reproductive function because of chemotherapy [6]. Second-look surgery is not routinely recommended after a complete clinical response to surgery followed by postoperative chemotherapy. However, it may be advised for patients with residual masses after initial treatment, especially if teratomatous elements are present or if tumor markers remain elevated [20]. In our case, the delayed presentation at an advanced stage was associated with a poor prognosis and metastasis despite surgical and chemotherapy treatment. Due to the aggressive behavior of these tumors and their significant mortality risk, preserving future fertility must be considered a secondary concern.

## Conclusions

Although the diagnosis of pregnancy should be considered in women of reproductive age with a positive β-hCG and sonographic findings, the possibility of a mixed ovarian germ cell tumor must also be included in the differential when a pelvic mass is present. Timely surgical intervention, when indicated, is critical, not only to improve prognosis and reduce the risk of rapid disease progression but also to maximize the chances of preserving fertility. Because early metastatic tumors are often too small to be detected on imaging, particularly ultrasound, a high index of suspicion must be maintained even when imaging appears normal. Prompt diagnosis and referral are key to improving cure rates in young women with ovarian masses. Although the long-term survival rate for ovarian germ cell malignancies is excellent with current treatment strategies, relapses after chemotherapy are associated with a poor prognosis, particularly in cases with higher grades and advanced stages. Proper counseling for the patient is crucial, including an explanation of prognostic factors and the likelihood of disease recurrence.
